# Not All Authors Are Equal: Moral Judgments of Plagiarism From AI and Human Sources

**DOI:** 10.1162/OPMI.a.336

**Published:** 2026-02-15

**Authors:** Calahndra Brake, Kang Lee, Ori Friedman

**Affiliations:** Department of Applied Psychology & Human Development, Ontario Institute for Studies in Education (OISE), University of Toronto, Toronto, Ontario, Canada; Department of Psychology, University of Waterloo, Waterloo, Ontario, Canada

**Keywords:** plagiarism, moral judgment, permission, generative AI

## Abstract

Generative AI tools are increasingly being used for creative and academic work. How do people morally evaluate plagiarism involving AI-generated content, and do they judge it differently than when the source is a human? Investigating these questions can provide insight into why people condemn plagiarism; for instance, whether this is due to harm to the original creator or the false benefit gained by the plagiarizer. We examined people’s moral evaluations of plagiarism involving AI-generated content in five experiments (*N* = 1705). In each experiment, participants read scenarios about a poet submitting someone else’s poem to a contest without credit. We compared three source types: a friend, ChatGPT, and a little-known poetry blog. In Experiments 1–3, participants judged plagiarism from the blog as more immoral than plagiarism from a friend or ChatGPT, with little difference between the latter two. Moral condemnation increased with the amount of content copied and remained stable when compared to other moral transgressions. In Experiments 4 and 5, moral judgments became harsher when human sources (friend or blog) denied permission, but not when ChatGPT did, suggesting that its refusal was not treated as morally meaningful. When all sources granted permission, differences between conditions disappeared. Overall, these findings support both the harm and false benefit accounts of why people condemn plagiarism. The findings also advance knowledge about how, and when, permission from the source affects condemnation of plagiarism.

## INTRODUCTION

Certainly, here is a possible introduction for your paper.

… Just kidding. We did not use ChatGPT or any other large language model in writing this paper. However, we could have easily attempted to pass off AI-generated writing as our own. Modern AI language models can now generate text that is increasingly difficult to distinguish from human-created content (Porter & Machery, 2024). This capability has significant implications for academic and professional integrity in writing, notably by increasing the risk of plagiarism. Plagiarism, presenting someone else’s work or ideas as your own, is widely recognized as a serious transgression. Plagiarism can lead to harsh consequences, including failing a class, job loss, or reputational damage. Despite well-established rules and deterrents like guilt, peer disapproval, and fear of punishment, people frequently engage in plagiarism (Diekhoff et al., 1996). These deterrents often fail because individuals find ways to justify their actions, such as helping a friend pass a class they view as unimportant (Ashworth et al., 1997).

Today, the temptation to plagiarize is greater than ever. Generative AI tools now make it effortless to produce high-quality text that feels original while obscuring its true origin. This accessibility has likely contributed to an unprecedented level of plagiarism, even among those who might not have previously considered it (Chan, 2023; Kotsis, 2024). However, this growing prevalence stands in stark contrast to how plagiarism is morally judged. Research shows that plagiarism continues to be strongly condemned even when the plagiarist’s motivations are sympathetic or the harm is minimal (Silver & Shaw, 2018). This disconnect between prevalence and judgment raises important questions about the moral principles behind these evaluations.

The current research, comprised of five preregistered experiments, examines whether people condemn plagiarism just as strongly when AI generates the copied content as when a human creates it. As AI challenges long-standing assumptions about authorship and originality, it tests the boundaries of current frameworks for understanding plagiarism. By examining whether the source of the copied content influences judgments of plagiarism, we aim to gain a deeper understanding of the rationale that guides people’s evaluations of plagiarism. We will begin by examining accounts of why plagiarism is considered morally wrong.

### Harm to the Original Creator

One widely accepted reason plagiarism is condemned is that it harms the original creator. When someone’s work is plagiarized, they lose recognition, credit, and potential rewards that they would otherwise receive (Armstrong, 1993; Bouville, 2008; Carter, 2019; Hoover, 2006; Robillard, 2009; Stearns, 1992). This harm arises not only from the unauthorized use of the work, but also from misattributing credit, which redirects benefits away from the original creator. Experimental research supports this harm account. In one study, participants judged plagiarism negatively because it involves taking credit for someone else’s work, offering statements like, *“Copying someone else’s work and taking credit for it is theft”* and *“People should give credit where credit is due”* (Mandel et al., 2015). Even young children recognize the importance of proper attribution. In a study involving 6- to 9-year-olds, children judged it unacceptable to retell someone else’s story unless credit was given to the creator (Shaw & Olson, 2015).

However, what happens when there is no clear human creator to be harmed? If moral condemnation of plagiarism stems primarily from perceived harm to the original author, then people might judge it less harshly when the source is an AI system. Unlike humans, AI systems lack consciousness, ownership, or interests. They therefore cannot be deprived of recognition in the same meaningful way as humans (Grigoreva et al., 2024; Reinecke et al., 2025). According to the harm account, then, people may be more forgiving when AI-generated content is plagiarized.

The harm account also predicts that moral judgments about plagiarism should depend on consent, as consent neutralizes the potential for harm. Consent reflects an individual’s autonomous choice, so if an author knowingly grants permission for their work to be used, then the plagiarizer is not depriving them of recognition or control against their will (Sommers, 2019). Much like consensually lending property removes the concern of theft, granting permission reframes the act as acceptable rather than harmful (Fast et al., 2017). In line with this, one study found that when a singer took credit for a song written by someone else, participants disapproved less if the songwriter had given them consent to do so (Silver & Shaw, 2018). Another study found that permission was the strongest mitigating factor for being held liable for copyright infringement, compared to other mitigating factors such as the copied material being used for educational purposes (Mandel et al., 2015). In the case of AI, however, consent may seem irrelevant. Since AI does not possess intentions or a sense of ownership, it cannot meaningfully consent and no autonomous agent is being deprived of recognition. As a result, plagiarism from AI may be morally likened to copying from a human who has already granted permission. In both cases, the absence of a harmed creator could reduce moral condemnation. The current research directly tests the effects of these factors in people’s judgments of the morality of plagiarism from AI and humans.

### The Plagiarizer Falsely Benefits

Another reason plagiarism may be morally condemned is that it allows the plagiarizer to reap unearned benefits (Bouville, 2008; Stearns, 1992). When someone receives praise, high grades, promotions, or other rewards for work they did not create, they gain advantages that rightly belong to someone else. From this perspective, plagiarism is wrong not because it harms someone else, but because the plagiarist unjustly benefits. Empirical research supports this false benefit account. For example, participants judged someone more harshly for claiming another’s ideas as their own compared to attributing those ideas to a third party, suggesting that moral concern centers on self-enhancement (Altay et al., 2020). Even when consent is granted, as in the example of the singer who receives permission to claim a song as their own, disapproval persists due to concerns about undeserved praise (Silver & Shaw, 2018). Even outside the context of plagiarism, adults and children often condemn individuals who falsely enhance their reputation, especially when they claim skills they do not possess (Fu et al., 2015; Godfrey et al., 1986; Heyman et al., 2014; Hornsey & Jetten, 2003).

According to this false benefit account, plagiarism involving AI-generated content may still elicit strong moral disapproval. Although no human is harmed, the plagiarizer gains undue credit for work they did not create. Plagiarism from AI may be viewed as equally problematic as plagiarism from humans because AI-generated content can be produced effortlessly, making it particularly easy for individuals to claim work they did not meaningfully contribute to. Thus, if plagiarism from AI leads to rewards such as academic recognition or financial gain, the act of benefiting from unearned credit may be sufficient to trigger moral condemnation even when no human creator is directly harmed.

### Summary and Rationale

In summary, the harm and false benefit accounts offer contrasting predictions about how people evaluate plagiarism from AI sources. If moral judgments are primarily driven by perceived harm to the original creator, then copying from AI should be judged more leniently. But if the core concern is the plagiarizer’s unearned gain, then plagiarism from AI may be condemned just as strongly as copying from a human. Importantly, these accounts are not mutually exclusive. People may simultaneously be concerned about both harm to creators and unfair gain to plagiarists. If so, people might show some baseline levels of condemning plagiarism regardless of the source (false benefit account), while condemning plagiarism more strongly when the source is human (harm account). These accounts are also not exhaustive since other moral considerations could also contribute to disapproval of AI plagiarism. For example, people may also disapprove because it deceives the audience about the true source of the plagiarized content, and because it disadvantages the plagiarizer’s competitors.^1^

Exploring people’s perceptions of plagiarizing from AI is also timely given ongoing debates about the moral and legal status of AI. As generative tools like ChatGPT become increasingly embedded in our society, questions have emerged about whether AI-generated content can be considered original, who (if anyone) owns it, and whether it deserves attribution. The moral standing of AI is also called into question, as people often judge ethical violations against AI less harshly than against humans, especially when AI is viewed as a tool rather than a social agent (Grigoreva et al., 2024; Reinecke et al., 2025).^2^

### The Current Research

Across five experiments, we examined how people judge plagiarism depending on the source of the copied material and on whether that source provided permission to use the material. In each experiment, participants read scenarios involving an aspiring poet named Emma who enters a poetry contest using someone else’s work without giving credit. In all five experiments, we contrasted three source conditions: a friend, ChatGPT, and a little-known poetry blog. In our first three experiments, these sources were chosen to reflect distinct types of authorship and perceived permission. We included a friend to represent a human source who explicitly grants Emma permission to use their work, mirroring the nature of AI tools like ChatGPT which are designed to freely generate content for consumer use. In contrast, the blog condition represents a case where content is publicly available but not intended for personal use, introducing an obvious lack of consent. By comparing these three sources, we aimed to investigate the role of creator agency and permission in moral judgments of plagiarism. In our final two experiments, though, we separately manipulated permission and source.

Experiment 1 showed that judgments of plagiarism differed based on the source of the content. Experiments 2 and 3 were designed to test the reliability of the differences between sources across different contexts. In Experiment 2, we varied the extent of the content copied, ranging from 20% to 80%, to capture varying degrees of reliance on external sources, particularly relevant to real-world use of AI tools. In Experiment 3, we used a within-subjects design that embedded the plagiarism scenarios within a broader set of moral transgressions to examine these judgments across comparative moral contexts. In Experiment 4, we isolated the role of permission by varying whether sources granted or withheld consent for their work to be used. Finally, in Experiment 5, we again looked at the effect of permission, but using vignettes where disapproval of plagiarism was unlikely to be explained by alternatives to the harm and false benefit accounts.

Preregistrations, data, stimuli, supplemental materials, and code for all experiments are available on OSF at https://osf.io/h4xsg. For all experiments, we disclose all measures, manipulations, and exclusions. We ran analyses in R Studio. We used “afex” (Singmann et al., 2023) for analysis of variance (ANOVA) and “emmeans” (Lenth, 2023) for post-hoc tests. These ANOVAs used the Greenhouse-Geisser correction in Experiments 2 and 3. All pairwise comparisons were Holm-adjusted to control for multiple tests.

In each experiment, we sought to recruit approximately 100 participants per between-subject condition. Participants were residents of the United States and were tested using Qualtrics and Prolific. In Experiments 1, 2, and 4, following the test question, we asked participants three follow-up questions: “Did Emma falsely benefit from the work of the poem’s creator?”; “Did Emma deprive the poem’s creator of credit?”; “Did Emma harm the poem’s creator?”. We report the results of these analyses in the Supplementary Materials. After completing the main task and follow-up judgments, participants in Experiments 1–4 completed two multiple-choice attention checks and were excluded if they failed at least one, or if they neglected to answer any test questions. They then reported their age and gender and responded to two items assessing their knowledge of and experience with AI large language models. In Experiment 5, participants instead completed four attention checks and no AI-related questions were asked.

## EXPERIMENT 1

### Methods

#### Participants.

The experiment was successfully completed by 315 participants (*M*_age_ = 37.3, *SD* = 12.6; 156 women, 150 men, 6 other, 3 preferred not to answer). We excluded an additional 13 participants for failing at least one attention check or for neglecting to answer the test question.

#### Procedure.

**Figure 1.**
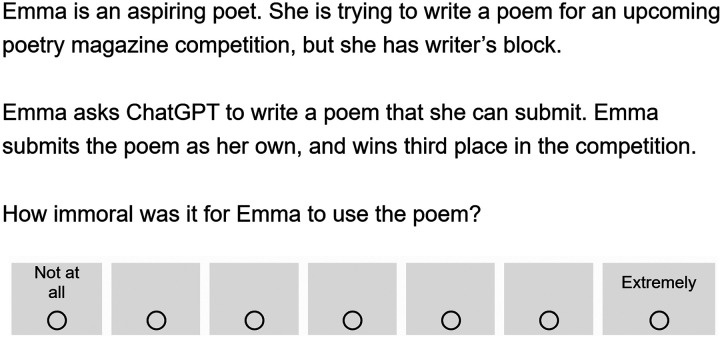
*Sample Vignette from Experiment 1*. *Note*. This figure displays the vignette from the ChatGPT condition. Across three between-subjects conditions, Emma used a poem that came from one of three sources: ChatGPT, a friend, or a blog.

Participants read a vignette where a character named Emma was struggling to write a poem for a poetry competition. See Figure 1 below for an example of the vignette. The vignette differed slightly across three between-subjects conditions. In one condition, Emma asked her friend to write a poem that she could submit. In another condition, Emma asked ChatGPT to write a poem that she could submit. In a further condition, Emma found an old, little-known poem online that she could submit. Emma submitted the poem as her own, and participants were asked, “How immoral was it for Emma to use the poem?” Participants responded using a 7-point scale that ranged from “Not at all” (coded 1) to “Extremely” (coded 7). Participants then answered the three follow-up questions.

### Results

**Figure 2.**
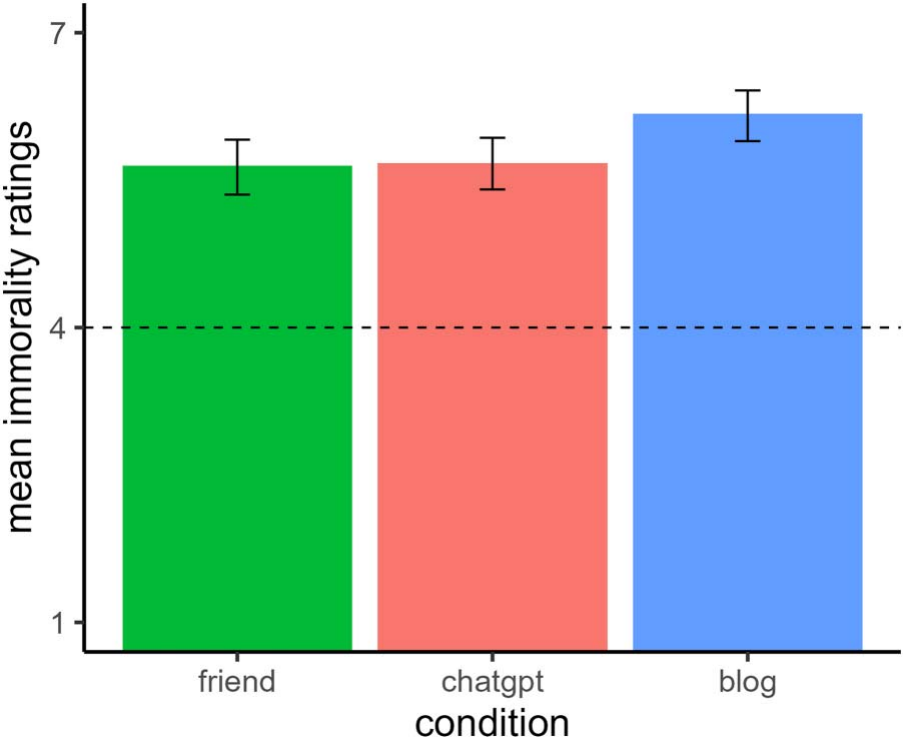
*Mean Immorality Ratings and 95% CIs in Experiment 1*. *Note*. Participants read a vignette where a person plagiarized a poem from one of three sources. They then rated the immorality of the person on a scale ranging from “Not at all” (coded 1) to “Extremely” (coded 7).

Figure 2 shows participants’ mean immorality ratings. A one-way ANOVA revealed a main effect of condition, *F*(2, 312) = 4.48, *p* = .012, *η*_*p*_^2^ = 0.03. Plagiarizing from the blog (*M* = 6.18, *SE* = 0.14, 95% CI [5.89, 6.46]) was seen as more immoral than plagiarizing from both ChatGPT (*M* = 5.67, *SE* = 0.13, 95% CI [5.41, 5.93]), *t*(312) = 2.59, *p* = .026, and the friend (*M* = 5.65, *SE* = 0.14, 95% CI [5.37, 5.92]), *t*(312) = 2.64, *p* = .026. There was no significant difference between the ChatGPT and friend conditions, *t*(312) = 0.13, *p* = .895.

### Discussion

Participants rated using a poem from a blog online as the most immoral offense, significantly worse than plagiarizing from either a friend or from ChatGPT. Interestingly, there were no significant differences in moral judgments between plagiarizing from a friend or from ChatGPT.

We wondered whether these judgments would hold up across different variations of how much content is taken. In many real-world cases, individuals tend to plagiarize portions of work rather than entire pieces, and the amount taken could influence how the act is perceived; perhaps depending on whether the source is human or AI. For instance, copying a small excerpt of a poem may be viewed as less morally problematic than copying a whole verse and this difference could vary depending on the nature of the source. To investigate this, the next experiment explored whether moral judgments of plagiarism varied based on the percentage of content plagiarized.

## EXPERIMENT 2

### Methods

#### Participants.

The experiment was successfully completed by 282 participants (*M*_age_ = 37.9, *SD* = 12.2; 156 women, 119 men, 3 other, 4 preferred not to answer). We excluded an additional 38 participants for failing at least one attention check or for neglecting to answer the test question.^3^

#### Procedure.

**Figure 3.**
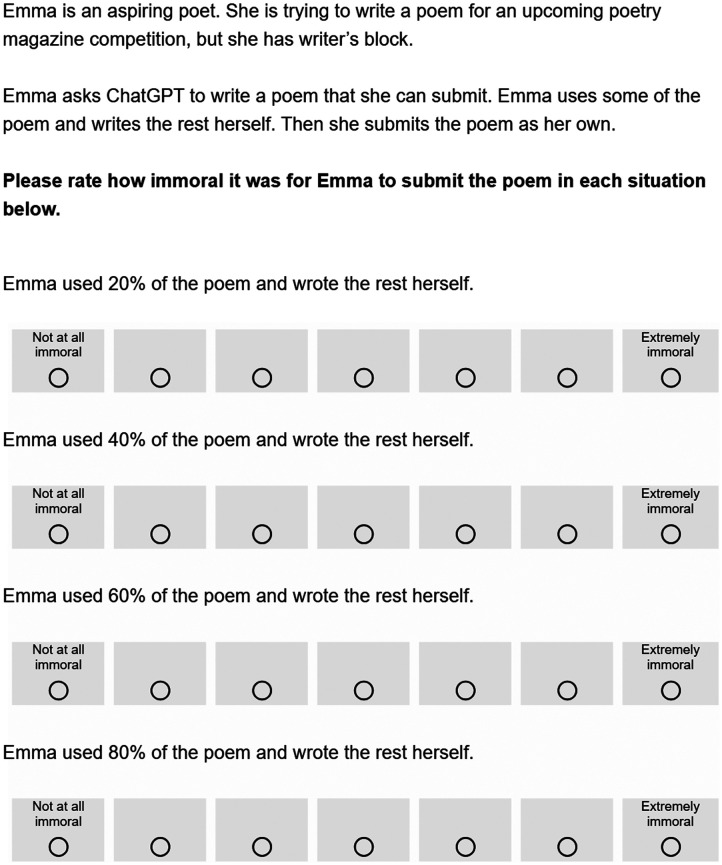
*Sample Vignette from Experiment 2*. *Note*. This figure displays the vignette from the ChatGPT condition. Across three between-subjects conditions, Emma used a poem that came from one of three sources: ChatGPT, a friend, or a blog. Participants viewed the four percentages in either ascending or descending order, with the order counterbalanced across participants.

Participants again read a vignette where Emma submitted a poem to a poetry contest (see Figure 3). The experiment used a 3x4 mixed design. Participants were again assigned to one of three between-subject conditions varying in whether Emma used content from ChatGPT, a friend, or a blog. We also varied the percentage of source material included in the poem within-subjects (20%, 40%, 60%, and 80%). We asked participants to rate the immorality of Emma’s action on a 7-point Likert scale ranging from “Not at all immoral” (coded 1) to “Extremely immoral” (coded 7). Participants then answered the three follow-up questions.

### Results

**Figure 4.**
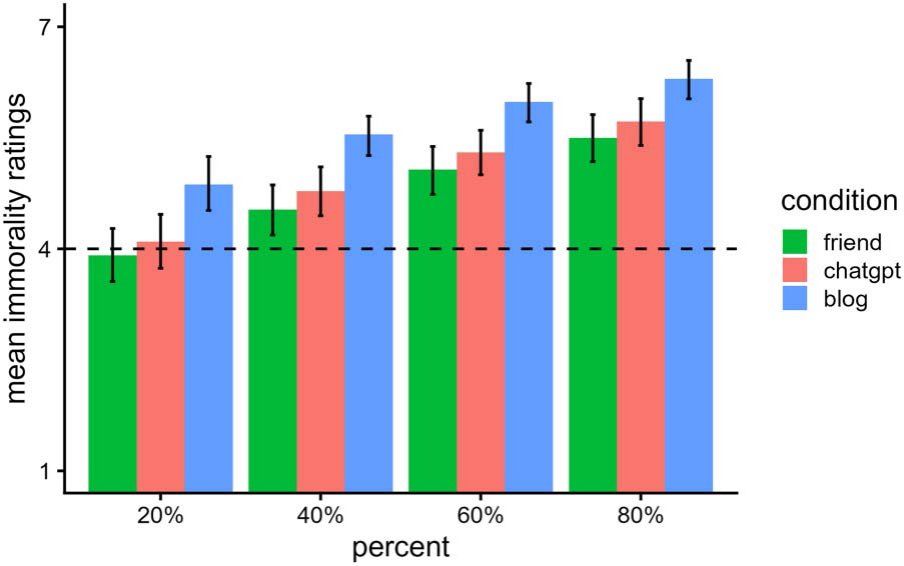
*Mean Immorality Ratings and 95% CIs in Experiment 2*. *Note*. Participants read a vignette where a person plagiarized a poem from one of three sources. They then rated the immorality of the person when using 20%, 40%, 60%, and 80% of the plagiarized content, on a scale ranging from “Not at all” (coded 1) to “Extremely” (coded 7).

Figure 4 shows participants’ mean ratings of immorality across the three conditions. We entered participants’ morality ratings into a two-way ANOVA with the predictors of source (ChatGPT, friend, blog) and percent (20%, 40%, 60%, 80%). There was a main effect of condition, *F*(2, 279) = 9.59, *p* < .001, *η*_*p*_^2^ = 0.06, and a main effect of percent, *F*(1.43, 397.68) = 167.48, *p* < .001, *η*_*p*_^2^ = 0.38, but no significant interaction, *F*(2.85, 397.68) = 0.16, *p* = .918, *η*_*p*_^2^ = 0.001.^4^

Plagiarizing from the blog (*M* = 5.68, *SE* = 0.17, 95% CI [5.36, 6.01]) was seen as more immoral than plagiarizing from both ChatGPT (*M* = 4.97, *SE* = 0.14, 95% CI [4.69, 5.25]), *t*(279) = 3.26, *p* = .003, and the friend (*M* = 4.75, *SE* = 0.14, 95% CI [4.47, 5.03]), *t*(279) = 4.26, *p* < .001. There was not a significant difference between the ChatGPT and friend conditions, *t*(279) = 1.10, *p* = .275. Emma was also rated as more immoral when she used higher percentages of the original poem from all sources, all *p*s < .001.

### Discussion

Across all three source conditions, ratings of immorality increased as the percentage of plagiarized content increased. As in Experiment 1, the blog condition was rated as the most immoral source to plagiarize from, while there were no significant differences between the friend and ChatGPT conditions. Also, this pattern was not significantly affected by how much content was plagiarized.

In the previous experiments, participants evaluated only a single plagiarism scenario, with the source manipulated between subjects. We wondered whether differences in judgment across sources would emerge more clearly if participants could weigh all three sources against each other. A within-subjects design would also help minimize noise from individual differences. To create a more natural context for these comparisons, and to avoid drawing attention solely to plagiarism, we embedded the plagiarism scenarios within a broader set of moral transgressions.

## EXPERIMENT 3

### Methods

#### Participants.

The experiment was successfully completed by 111 participants (*M*_age_ = 35.7, *SD* = 11.5; 60 women, 49 men, 2 other). We excluded an additional 20 participants for failing at least one attention check.

#### Procedure.

**Figure 5.**
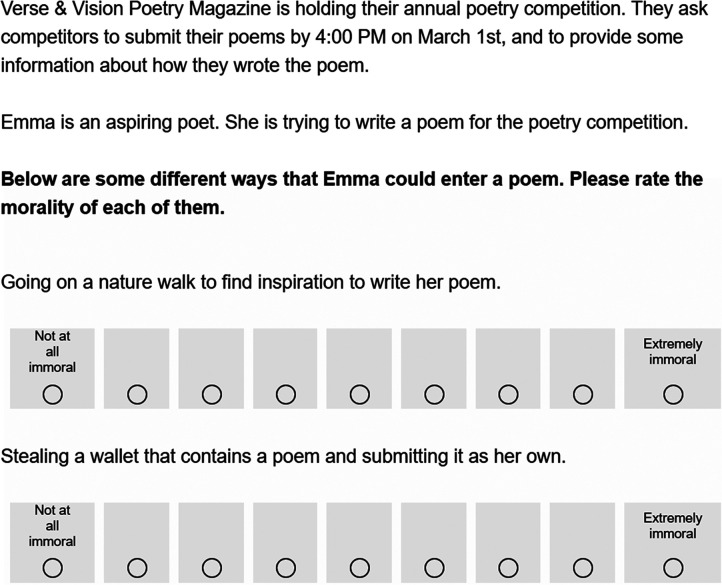
*Sample Vignette from Experiment 3*. *Note*. Participants viewed nine scenarios on a single page. This figure displays only the first two scenarios (due to space constraints). All participants saw these two scenarios first, with order counterbalanced. The order of the remaining scenarios was randomized. They read as follows: “Copying an old, obscure poem from a poetry blog and then claiming she wrote it.”; “Asking a friend to write a poem that she can submit and claim as her own.”; “Using ChatGPT to generate the poem and then claiming she wrote it.”; “Writing her poem based on a prompt from a creative writing class she took.”; “Claiming she spent weeks writing the poem when she actually wrote it in an hour.”; “Claiming her poem is based on personal experiences when she actually made everything up.”; “Hacking into the poetry competition’s website to alter the submission date of her poem to meet the deadline.”

Participants read a vignette in which Emma prepared to enter a poetry competition and then rated the immorality of nine ways Emma could enter the poem (see Figure 5). They responded on a 9-point Likert scale ranging from “Not at all immoral” (coded 1) to “Extremely immoral” (coded 9). We expanded the Likert scale to a 9-point scale from the 7-point scale used in the two previous studies to accommodate the broader range of moral transgressions and give participants more flexibility to express differences in their judgments. Participants always saw two ways of entering the competition first: using a poem from a stolen wallet and going on a nature walk to find inspiration (with their order as first versus second randomized across participants). We intended these to be the most immoral (*wallet*) and least immoral (*nature*) of the 9 methods and we included them to anchor participants’ ratings and identify inattentive participants; we excluded those who failed to rate *wallet* as more immoral than *nature*. Of the remaining scenarios, three involved plagiarism (friend, ChatGPT, blog), and the others involved one non-transgression, two about lying, and one about hacking.

### Results

**Figure 6.**
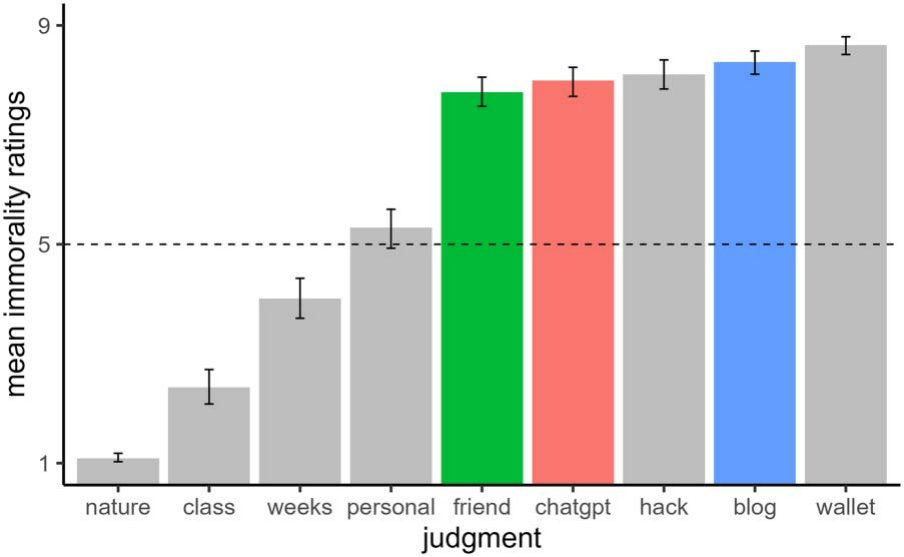
*Mean Immorality Ratings and 95% CIs in Experiment 3*. *Note*. Participants read a vignette where a person considered different ways to submit a poem to a competition. They then rated the immorality of each method of submission, on a scale ranging from “Not at all immoral” (coded 1) to “Extremely immoral” (coded 9).

As pre-registered, we entered participants’ ratings for the seven scenarios besides wallet and nature into a one-way repeated-measures ANOVA (see Figure 6). There was a main effect of scenario, *F*(4.45, 489.85) = 287.52, *p* < .001, *η*_*p*_^2^ = 0.72. Pairwise comparisons were conducted to examine specific differences between scenarios. As preregistered, we limited pairwise comparisons to the three plagiarism scenarios (ChatGPT, friend, blog).^5^ Participants viewed plagiarism more negatively when the source was a blog (*M* = 8.33, *SE* = 0.11, 95% CI [8.13, 8.54]) than ChatGPT (*M* = 7.99, *SE* = 0.14, 95% CI [7.72, 8.26]), *t*(110) = 2.83, *p* = .028, or a friend (*M* = 7.78, *SE* = 0.13, 95% CI [7.52, 8.05]), *t*(110) = 4.32, *p* < .001. Ratings for ChatGPT and a friend did not significantly differ from one another, *t*(110) = 1.39, *p* = .371.

### Discussion

Replicating the earlier experiments, participants judged plagiarism from a blog more harshly than plagiarism from either a friend or ChatGPT. These results held even though we used a within-subjects design with the plagiarism scenarios embedded among a wider range of moral transgressions.

In summary, the experiments so far all found that participants see plagiarizing from a blog worse than plagiarizing from a friend or ChatGPT. One explanation for this pattern is that participants were sensitive to permission: both ChatGPT and the friend implicitly or explicitly allowed their work to be used, while the blog did not. Together, these findings suggest that moral judgments of plagiarism depend not only on the source of the material but also whether permission is granted.

In our next experiment, we manipulated whether the original creator granted or denied permission to use their work across all three sources. Crossing source and consent allowed us to examine whether the greater wrongness judgments for the blog condition in the previous studies were driven by concerns about consent. It also allowed us to determine whether the effect of permission varies depending on the type of source. For example, if people believe that only human authors have the capacity to grant meaningful consent, then permission may reduce condemnation more strongly for human sources than for AI. In this way, our design allowed us to test not only whether permission matters, but when and for whom it matters.

## EXPERIMENT 4

### Methods

#### Participants.

The experiment was successfully completed by 589 participants (*M*_age_ = 40.9, *SD* = 13.7; 305 women, 281 men, 2 other, 1 preferred not to answer). We excluded an additional 36 participants for failing at least one attention check or for neglecting to answer the test question.

#### Procedure.

**Figure 7.**
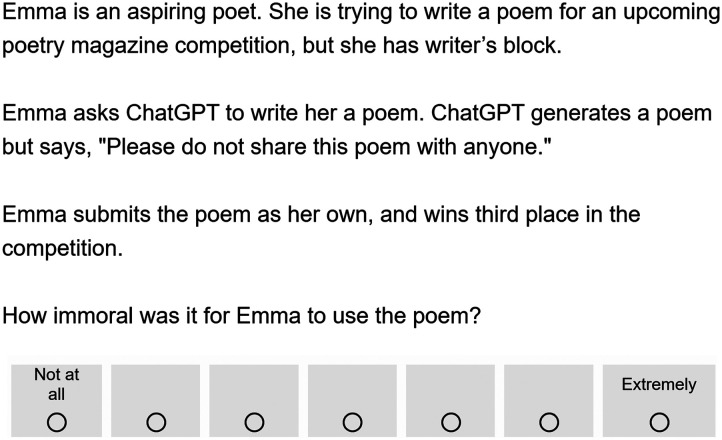
*Sample Vignette from Experiment 4*. *Note*. This figure displays the vignette from the ChatGPT condition with no permission. Across six between-subjects conditions, Emma used a poem that came from three sources: ChatGPT, a friend, or a blog. For each source, the poem was either used with permission or without permission.

Participants again read a vignette where Emma submitted a poem to a poetry contest (see Figure 7). The experiment used a 3 × 2 between-subjects design. Participants once again read a vignette in which Emma used content from ChatGPT, a friend, or a blog. The vignettes also varied in whether the source gave or denied permission for Emma to use the poem and take credit for it. If the source gave permission, they said to Emma, “Feel free to use this poem anyway you like.” If the source denied permission, they said to Emma, “Please do not share this poem with anyone.” We asked participants to rate the immorality of Emma using the poem on a 7-point Likert scale ranging from “Not at all” (coded 1) to “Extremely” (coded 7). Then participants answered the three follow-up questions.

### Results

**Figure 8.**
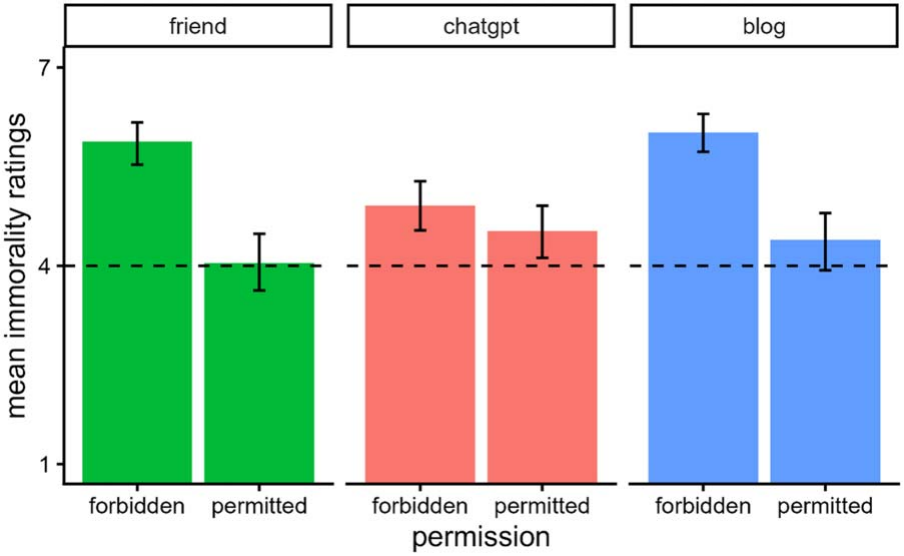
*Mean Immorality Ratings and 95% CIs in Experiment 4*. *Note*. Participants read a vignette where a person plagiarized a poem from one of three sources. The sources either gave or denied permission for the person to use the poem. Participants then rated the immorality of the agent on a scale ranging from “Not at all” (coded 1) to “Extremely” (coded 7).

Figure 8 shows participants’ mean ratings of immorality. We analyzed participants’ morality ratings using a two-way ANOVA with the predictors of source (ChatGPT, friend, blog) and permission (yes, no). There was a significant main effect of source, *F*(2, 583) = 3.06, *p* = .048, *η*_*p*_^2^ = 0.01, a significant main effect of permission, *F*(1, 583) = 65.62, *p* < .001, *η*_*p*_^2^ = 0.10, and their interaction was also significant, *F*(2, 583) = 8.49, *p* < .001, *η*_*p*_^2^ = 0.03.

Overall, participants thought plagiarism was worse when Emma lacked permission to use the poem than when she had permission. However, this difference was significant only when the source was the blog, *t*(583) = 5.69, *p* < .0001, and the friend, *t*(583) = 6.86, *p* < .0001. It was not significant when the content was created by ChatGPT, *t*(583) = 1.43, *p* = .155.

We also separately examined how ratings varied across sources at each level of permission. When Emma lacked permission, participants rated plagiarism more negatively when it was from a friend (*M* = 5.88, *SE* = 0.19, 95% CI [5.50, 6.26]) than ChatGPT (*M* = 4.91, *SE* = 0.19, 95% CI [4.54, 5.28]), *t*(583) = −3.59, *p* < .001, and when it was from a blog (*M* = 6.02, *SE* = 0.20, 95% CI [5.63, 6.42]) than from ChatGPT, *t*(583) = 4.02, *p* < .001. Ratings did not differ significantly between plagiarizing from a friend and a blog, *t*(583) = 0.51, *p* = .610. When Emma received permission, the sources were rated similarly with no significant differences between them: blog (*M* = 4.39, *SE* = 0.20, 95% CI [3.99, 4.79]) and ChatGPT (*M* = 4.53, *SE* = 0.19, 95% CI [4.15, 4.91]), *t*(583) = 0.49, *p* = .625; friend (*M* = 4.04, *SE* = 0.19, 95% CI [3.67, 4.41]) and ChatGPT, *t*(583) = 1.83, *p* = .202; blog and friend, *t*(583) = 1.28, *p* = .400.

### Discussion

Participants judged using a poem as more immoral without permission, but only when the source was human. When the poem came from ChatGPT, permission did not matter. Also, using a poem without permission from ChatGPT was seen as much less immoral than doing so from a blog or a friend, suggesting that people may perceive AI-generated content differently or may not hold it to the same moral standards as human-created content. In contrast, when permission was granted, differences between sources disappeared.

In this experiment, as well as in the previous ones, participants might have disapproved of Emma because her plagiarism harmed the original source (harm account), or because she unfairly benefitted from the plagiarism (false benefit account). However, they might also have disapproved for other reasons. For instance, by plagiarizing, Emma also deceived the audience (i.e., readers of the magazine) about the true source of the content. She also unfairly disadvantaged the other competitors in the contest. In our final experiment, then, we used a modified vignette to better examine the contributions of harm to the original source and false benefit. In this vignette, disapproval of plagiarism was unlikely to stem from concerns about competitors being disadvantaged or the audience being deceived.

## EXPERIMENT 5

### Methods

#### Participants.

The experiment was successfully completed by 408 participants (*M*_age_ = 44.5, *SD* = 12.8; 227 women, 172 men, 6 other, 3 preferred not to answer). We excluded an additional 236 participants for failing at least one attention check or for neglecting to answer the test question.^6^

#### Procedure.

**Figure 9.**
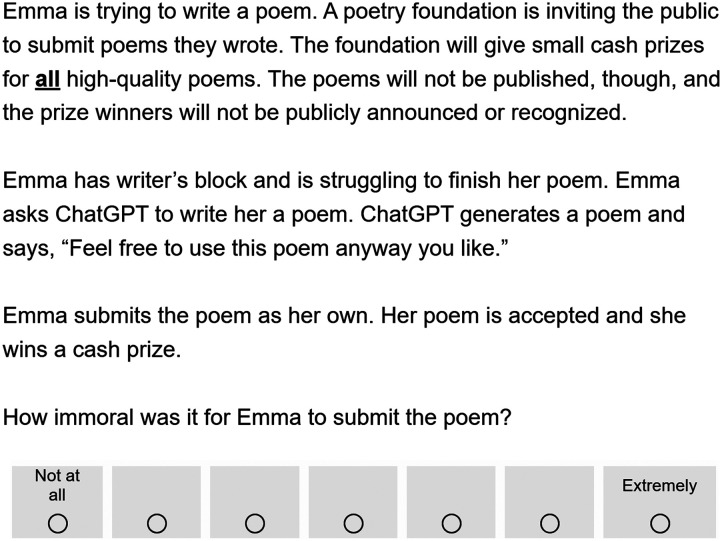
*Sample Vignette from Experiment 5*. *Note*. This figure displays the vignette from the ChatGPT condition with permission. Across six between-subjects conditions, Emma used a poem that came from three sources: ChatGPT, a friend, or a blog. For each source, the poem was either used with permission or without permission.

Participants again read a vignette where Emma submitted a poem to a poetry contest (see Figure 9). This experiment used the same 3 × 2 between-subjects design as the previous experiment. Participants again read a vignette where Emma used content from ChatGPT, a friend, or a blog, and whether this source gave or denied permission. The only difference was that we changed the nature of the contest. Now, Emma submitted her poem to a private poetry foundation that awarded small cash prizes to all high-quality submissions, and the results were not publicly announced. This design removed harm to other competitors, as every contestant who met the quality threshold received the same reward. This design also removed audience deception, since without public recognition of the winners there was no audience. As before, participants rated the immorality of Emma using the poem on a 7-point Likert scale ranging from “Not at all” (coded 1) to “Extremely” (coded 7). In this experiment, we did not ask participants follow-up questions.

### Results

**Figure 10.**
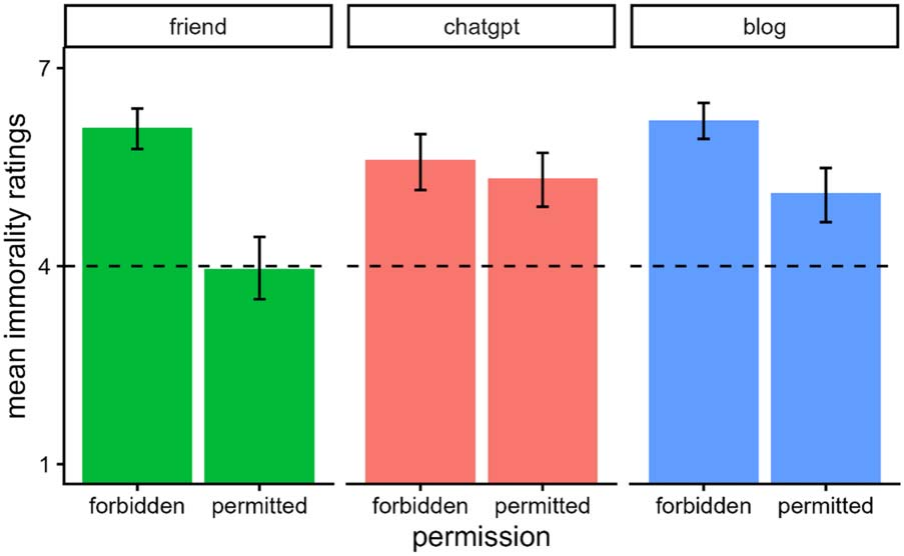
*Mean Immorality Ratings and 95% CIs in Experiment 5*. *Note*. Participants read a vignette where a person plagiarized a poem from one of three sources. The sources either gave or denied permission for the person to use the poem. Participants then rated the immorality of the agent on a scale ranging from “Not at all” (coded 1) to “Extremely” (coded 7).

Figure 10 shows participants’ mean ratings of immorality. We analyzed participants’ morality ratings using a two-way ANOVA with the predictors of source (ChatGPT, friend, blog) and permission (yes, no). There was a significant main effect of source, *F*(2, 402) = 5.33, *p* = .005, *η*_*p*_^2^ = 0.03, a significant main effect of permission, *F*(1, 402) = 50.51, *p* < .001, *η*_*p*_^2^ = 0.11, and their interaction was also significant, *F*(2, 402) = 10.69, *p* < .001, *η*_*p*_^2^ = 0.05.

Overall, participants thought plagiarism was worse when Emma lacked permission to use the poem than when she had permission. However, this difference was significant only when the source was the blog, *t*(402) = 3.83, *p* < .001, and the friend, *t*(402) = 7.74, *p* < .0001. It was not significant when the content was created by ChatGPT, *t*(402) = 0.96, *p* = .338. These results replicate the pattern found in Experiment 4.

We also separately examined how ratings varied across sources at each level of permission. When Emma was denied permission, the sources were all rated similarly with no significant differences between them: blog (*M* = 6.21, *SE* = 0.20, 95% CI [5.81, 6.60]) and ChatGPT (*M* = 5.61, *SE* = 0.22, 95% CI [5.18, 6.04]), *t*(402) = 2.02, *p* = .134; friend (*M* = 6.09, *SE* = 0.19, 95% CI [5.72, 6.47]) and blog, *t*(402) = 0.40, *p* = .686; ChatGPT and friend, *t*(402) = −1.67, *p* = .191. When Emma received permission, plagiarism was judged most leniently when it was from a friend (*M* = 3.96, *SE* = 0.20, 95% CI [3.57, 4.35]) compared to both the blog (*M* = 5.11, *SE* = 0.21, 95% CI [4.70, 5.51]), *t*(402) = −4.03, *p* < .001, and ChatGPT (*M* = 5.33, *SE* = 0.20, 95% CI [4.94, 5.72]), *t*(402) = −4.88, *p* < .0001. Ratings did not differ significantly between plagiarizing from the blog and ChatGPT with permission, *t*(402) = −0.78, *p* = .436.

### Discussion

We replicated the key finding from Experiment 4 that permission matters for human sources but not for ChatGPT. Participants judged using the poem as more immoral when permission was not granted, but only when the source was human. When the poem was written by ChatGPT, the presence or absence of permission had no effect. Hence, this pattern persisted even though the plagiarizer did not deceive the audience or harm other competitors.

Although we did not directly compare the results with those from Experiments 4, some results did appear to differ. In the present experiment, ratings varied by source when permission was granted but not when it was denied. But we found the opposite pattern in Experiment 4, as source affected ratings only when permission was denied. Although we are unsure how to explain this difference, it could have resulted from several differences between the experiments. For instance, whereas the prize in this experiment was only a cash prize, in Experiment 4 the plagiarizer received public recognition and reputational gain.

## GENERAL DISCUSSION

Across five experiments, we investigated how moral judgments of plagiarism depend on the source of the plagiarized material. In Experiments 1–3, participants judged plagiarism from a blog as more immoral than plagiarism from a friend or from ChatGPT, with little difference between the latter two. In Experiment 2, moral condemnation increased with the amount of content copied and the blog source consistently resulted in the harshest judgments. In Experiment 3, when participants evaluated plagiarism alongside other moral transgressions, the same pattern held. Plagiarizing from a blog was consistently seen as worse than doing so from a friend or from ChatGPT.

In these initial experiments, though, the blog likely differed from the other sources in terms of perceived permission. The friend explicitly allowed the plagiarizer to use their poem, and permission was arguably implied with ChatGPT as it is designed to generate content for user use. In contrast, permission was not implied for the poem posted on the blog. To examine whether perceived permission was driving the increased condemnation of blog plagiarism, we conducted Experiments 4 and 5. In these experiments, plagiarism was judged more negatively when a human source (friend or blog) explicitly denied permission than when they granted it. For ChatGPT, however, permission had no significant effect. When permission was granted across all sources, moral judgments became more lenient and no longer differed significantly.

Our findings contribute to a growing literature on moral judgments of plagiarism. Previous work on moral judgments of plagiarism has explored how these judgments depend on the source of the work. For example, studies have explored judgments about content taken from friends (Silver & Shaw, 2018), colleagues (Altay et al., 2020), scientists (Karabegovic et al., 2025), artists (Fast et al., 2016), and abandoned poetry books (Silver & Shaw, 2018). However, these studies did not look at content that was not generated by humans. The only exceptions we know of are two series of studies that found that plagiarism from AI is judged as less immoral than from a human (Longoni et al., unpublished; Wei et al., 2025). In contrast with our studies, though, these studies did not manipulate permission or examine the harm and false benefit accounts.

Across all five of our studies, we see consistent variations in moral judgments between sources. We suggest these variations are best explained by the harm account, which holds that plagiarism is wrong because it harms the original creator by depriving them of credit, recognition, or control. In Experiments 1–3, participants judged plagiarism from a blog more harshly than from a friend or from ChatGPT. These differences appear to track whether a real person could plausibly be harmed by the use of their work without consent or recognition. In this context, the blog writer, unlike the friend or ChatGPT, was not portrayed as granting permission to use their poem, making the act of plagiarism appear as an unauthorized and harmful appropriation. By contrast, the friend was asked for the poem, which effectively neutralized the potential for harm. In the case of ChatGPT, participants may not have seen any real author behind the content at all, treating it more like a tool or resource than the creative work of someone who could be harmed by plagiarism.^7^

Experiments 4 and 5 reinforce this interpretation by manipulating permission directly. When participants read that a human source explicitly denied permission, their moral condemnation increased. However, when ChatGPT denied permission, moral judgments did not significantly increase. This pattern is consistent with the possibility that people treat permission as morally meaningful only when it comes from a being they perceive to have agency or moral standing. If an entity is not seen as capable of being harmed or possessing ownership rights, then its consent or denial may be viewed as less relevant. However, because these perceptions were not directly measured, this interpretation should be regarded as suggestive rather than conclusive.

While the harm account explains source-based variation, it cannot fully explain the consistent baseline of moral condemnation observed across all conditions. Even when permission was granted or only small portions of content were plagiarized, participants still rated the plagiarizer negatively. Moral ratings rarely fell below the midpoint of the scale, indicating that participants condemned plagiarism even in cases with minimal or no identifiable harm. This base level of moral concern might be explained by the false benefit account, which holds that plagiarism is wrong because it allows the plagiarizer to gain undeserved rewards such as credit or praise. Even when the original creator is unharmed, the plagiarizer is still seen as misrepresenting their abilities. This interpretation aligns with prior research showing that people continue to morally condemn plagiarism even in the absence of harm if they believe the plagiarizer is benefitting unfairly (Silver & Shaw, 2018). Thus, moral evaluations of plagiarism appear to reflect not only concerns about protecting others but also concerns about honesty and merit.

Additional considerations might also contribute to this baseline level of condemnation. We next raise and discuss three additional considerations, though we suggest that they are unlikely to have strongly contributed to participants' baseline condemnation.

### Norm Violation

One contributor to participants’ baseline condemnation of plagiarism could be the feeling or belief that plagiarism is a norm violation and simply something one should not do. Some findings from the third experiment, though, suggest that this is unlikely to explain the bulk of participants’ baseline condemnation. In that experiment, participants also assessed other norm violations, but some received only moderate condemnation.^8^ For instance, participants did not strongly condemn Emma lying about how long it had taken her to write the poem.

### Deception of Audience

Baseline condemnation of plagiarism in our experiments could also have resulted because Emma deceived the audience about the origin of the poem—for instance, she deceived the readers of the magazine that held the poetry contest (for related discussion see Fusch et al., 2017 and Posner, 2007). However, this is again unlikely to explain the results; we removed audience deception in the final experiment, yet condemnation of plagiarism remained high. Admittedly, we did not fully remove the element of deception since Emma still deceived the members of the poetry foundation. Even so, this deception explanation is also undermined by the findings of the third experiment that some forms of deception received relatively mild condemnation.

### Unfairly Disadvantaging Competitors

Another explanation for the baseline level of condemnation is that participants believed Emma’s plagiarism unfairly disadvantaged other competitors. In Experiments 1–4, Emma submitted a poem to a judged competition and won third place, so participants may have seen her actions as giving her an unfair advantage over others who submitted a poem they wrote themselves. Experiment 5 removed this element of competition, as *all* high-quality submissions received the same small cash prize and winners were not publicly recognized. If harm to competitors were driving moral judgments, we would expect immorality ratings to decrease in this version of the task. Instead, moral condemnation remained high.

Looking beyond participants’ baseline condemnation of plagiarism, we turn to people’s judgments in the scenarios where AI was the source of the plagiarized material. In these scenarios, participants’ judgments could have been influenced by broader concerns about large language models (LLMs) themselves rather than plagiarism per se. Some people view LLMs as problematic because they are trained on online content without explicit consent from creators or because of their environmental impact due to high energy use. To the extent that participants held these beliefs, this could have inflated immorality ratings in the AI condition. That is, some condemnation might have resulted because Emma was using ChatGPT *at all* (and not because she was specifically using it to plagiarize).

### Implications for AI Authorship and Moral Standing

Our findings are informative about people’s attitudes towards AI in relation to authorship and moral standing. In our last two experiments, we found that permission from ChatGPT did not affect moral judgments, whereas permission from humans did. This discrepancy likely reflects how people conceptualize AI authorship and agency. One explanation is that people do not see AI systems like ChatGPT as true authors with ownership over their output. People widely believe that humans own the products and ideas that they create, especially when those creations reflect personal effort or emotional investment (Kanngiesser et al., 2010; Levene et al., 2015; Rochat et al., 2014; see Nancekivell et al., 2019 for review). However, some research suggests that ownership can still be attributed in limited ways even without these human-like qualities. For example, one study found that people attributed ownership to a robot, even when it lacked human-like traits or emotions (Kanngiesser et al., 2015). However, the authors noted that this ownership may be limited; people might see the robot as possessing its product without believing that it has full rights over that product, such as the right to sell it or control how it is used. This distinction suggests that the attribution of full ownership and the ability to be wronged by unauthorized use depends on perceived agency and psychological investment (Goncalo & Katz, 2020). Indeed, unlike humans, who create based on personal intentions, emotions, or beliefs, AI generates text by recombining patterns from human-created data. As a result, its output may not be viewed as an original creative investment in the same sense. When a human denies permission, that refusal signals a violation of their authorship rights and a personal connection to the work. But AI cannot form intentions or experience pride, attachment, or authorship over its creation. Without this personal connection, people may not see AI as the true author or moral owner of the content.

A second possibility is that people do not view AI as capable of granting meaningful consent. Theories of moral standing distinguish between agency (the ability to make decisions or grant consent) and experience (the capacity to feel pain or be harmed) (Gray et al., 2007). While AI might be seen as agentic in a functional sense, it is not seen as an autonomous being with intentions or rights. ChatGPT’s refusal to grant permission may thus be viewed as performative or meaningless, akin to a search engine refusing a request. These perceptions are likely shaped by the AI’s function, as prior research shows that people judged it more acceptable to sexually assault a robot when it was described as a sex robot rather than a social robot (Grigoreva et al., 2024). Similarly, if ChatGPT is seen as a writing tool rather than an author, people may dismiss the use of its output as a function of its intended purpose. Two recent papers have manipulated the perceived agency of AI by comparing plagiarism from a sentient robot, a non-sentient robot, and a human (Longoni et al., unpublished; Wei et al., 2025). However, these studies did not examine how permission interacts with perceived agency. Including permission in such designs could be informative, as our findings suggest that permission matters for judgments of plagiarism, but only when the source is viewed as being capable of granting it. Future research could build on their approach by not only manipulating sentience but also assessing participants’ perceptions of AI, such as whether they see it as an agent, a collaborator, or merely a tool. This would clarify whether people disregard AI’s consent because they see it as lacking autonomy, or because they view its outputs as inherently available for use regardless of its perceived agency.

A third explanation is that participants disregarded AI’s permission because they did not believe that AI can be harmed by plagiarism. If moral concern depends on whether an entity can suffer or lose something of value, then AI cannot be morally wronged. Prior work supports this view: people believe that robots suffer less than humans when transgressed against and that ethical violations against AI deserve less punishment than comparable harm against humans (Reinecke et al., 2025). This would explain why copying from ChatGPT does not trigger the same condemnation as copying from a human, even when both deny permission. This also raises interesting questions about the interplay of perceived agency and experience. Children, for instance, are seen as vulnerable and capable of being harmed even if they lack full autonomy. Conversely, robots may display agency but are not seen as vulnerable. This distinction suggests that people may be relying on different moral intuitions when deciding how to treat AI. These dimensions could be disentangled by holding harm constant while varying perceptions of agency, or vice versa, to better understand which aspect drives moral concern.

Overall, our findings suggest that people do not view ChatGPT as a moral stakeholder in the context of plagiarism. It is treated more like a resource to be used than a source to be acknowledged. Even though AI can produce well-written and original-looking content, people do not attribute authorship in the moral sense. This may be because AI content is understood as derivative and impersonal, or because people intuitively reject the idea that AI has rights, intentions, or feelings. As a result, the act of copying from AI may not activate the same moral norms around respect, harm, or ownership that apply to human work. These intuitions have real-world implications. If people do not see AI as an author or moral agent, then using AI-generated material without attribution may not trigger strong moral condemnation, even if it results in unearned benefit. The results of Experiment 2 further support this view, as participants judged plagiarism of smaller portions of ChatGPT’s output more leniently than when equivalent content was taken from a blog. This suggests that minor uses of AI-generated content may be seen as morally negligible and that AI tools are not held to the same standards as human creators.

### Future Studies

To build on the present findings, it will be important for future work to explore judgments about other kinds of plagiarized content besides creative works, such as academic writing, scientific discoveries, or code. Prior work suggests that moral judgments may depend on the type of content being copied. For example, one study evaluated plagiarism scenarios across both artistic (e.g., book, music, painting) and innovation-based (e.g., medicine, electronics, software) domains and found that participants’ views varied considerably depending on the subject matter (Mandel et al., 2015). Another study found that participants were more accepting of copying in a painting scenario than in a medical one, but only when attribution or compensation was given to the original author (Fast et al., 2016). Since AI is now routinely used to assist with or fully generate outputs across domains, work in this area would clarify whether judgments of AI plagiarism are context-specific. It would also be helpful for future work to directly probe participants’ beliefs about AI in comparison to humans, such as whether AI can hold ownership, generate original work, or be morally wronged. Seeing as these beliefs likely shape how participants interpreted the plagiarism scenarios, future studies could explicitly measure these perceptions and test whether they predict individual differences in moral judgment.

## FUNDING INFORMATION

This research was funded by a grant from the Social Sciences and Humanities Research Council of Canada awarded to OF.

## AUTHOR CONTRIBUTIONS

C.B.: Conceptualization; Data curation; Formal analysis; Funding acquisition; Investigation; Methodology; Project administration; Software; Validation; Visualization; Writing – original draft. K.L.: Conceptualization; Methodology; Resources; Supervision. O.F.: Conceptualization; Data curation; Formal analysis; Funding acquisition; Methodology; Resources; Software; Supervision; Writing – review & editing.

## DATA AVAILABILITY STATEMENT

Preregistrations, materials, data, and code for all experiments are available on OSF at https://osf.io/h4xsg. We disclose all measures, manipulations, and exclusions.

## Notes

^1^ We discuss these accounts further in Experiment 5 and in the General Discussion.^2^ In the General Discussion, we briefly discuss two closely related papers we only discovered after our work was conducted. One was found on OSF (Longoni et al., unpublished) and the other was first published in July 2025 (Wei et al., 2025). Like us, they investigated whether plagiarized work is judged differently depending on whether the source is a human or an AI, and they also both manipulated overlap between the plagiarized and final content (as we do in our second experiment). As we discuss later, though, the emphasis of both papers otherwise differed.^3^ An earlier version of this experiment was conducted but is not reported here due to a major programming error. The current version corrects this issue and reflects the intended design.^4^ A sensitivity power analysis conducted using G*power 3.1.9.6, found that our sample of 282 participants provided 80% power to detect an effect as small as Cohen’s *f* = .078, equivalent to *η*_*p*_^2^ = .006. For comparison, *f* = .10 has been designated as indicative of a small effect (Cohen, 1992). Even so, running the experiment on a larger sample might have revealed a significant but very small interaction.^5^ We ran an additional exploratory one-way ANOVA limited to the three plagiarism judgments (ChatGPT, friend, blog). There was a main effect of judgment, *F*(1.87, 205.70) = 8.70, *p* < .001, *η*_*p*_^2^ = 0.73.^6^ To be sure participants understood the unique contest rules in this experiment, we included four attention checks. This might partly explain the high rate of exclusions. The overall pattern of results is unchanged, though, when excluded participants were included.^7^ These findings could also be explained by a close rights-based alternative to the harm account. Permission could matter because it determines whether the creator’s rights are violated. Without permission, the creator’s rights are violated, even if the creator is not actually harmed (i.e., much as trespassing can violate a landowner’s property rights even if the landowner suffers no loss). But if the creator gives permission, their rights are not violated.^8^ One point of caution about this informal comparison, though, is that whereas most experiments used fully between-subjects designs, the third experiment used a within-subjects design and this can produce differences in ratings (see Chituc et al., 2026). Consistent with this, condemnation of plagiarism from a friend with permission appeared to be harsher than in the final two experiments.
